# Follicular development impairment in polyendocrine metabolic ovarian syndrome: multidimensional pathophysiological networks and frontier therapeutic targets

**DOI:** 10.3389/fimmu.2026.1879713

**Published:** 2026-10-07

**Authors:** Qiucheng Jia, Shuli Zhang, Lixin Zhang, Guangzong Zhao, Qi Zhu, Hongshan Ge

**Affiliations:** A Reproductive Medicine Center, The Affiliated Taizhou People’s Hospital of Nanjing Medical University, Taizhou School of Clinical Medicine, Nanjing Medical University, Taizhou, China

**Keywords:** circadian rhythm, epigenetic regulation, follicular development disorder, gut-brain-ovary axis, immune cell infiltration, metabolic network, neuroendocrine, organelle stress

## Abstract

Polyendocrine metabolic ovarian syndrome (PMOS) is a prevalent endocrine-metabolic disorder characterised by follicular arrest, anovulation, and elevated androgen levels. This review transcends single-dimensional analyses of PMOS pathology from neuroendocrine or metabolic perspectives, proposing an integrated multi-layered network model to systematically elucidate the pathological mechanisms underlying follicular arrest. Post-pubertal dysfunction of the hypothalamic-pituitary-ovarian axis interacts with systemic metabolic dysregulation, jointly establishing an ovarian microenvironment characterised by hyperandrogenism, hyperinsulinaemia, and chronic inflammation. This environment further induces self-reinforcing vicious cycles within follicles, manifesting as impaired granulosa-oocyte communication, immunometabolic reprogramming (e.g., M1 macrophage polarisation), and organelle stress (including mitochondrial dysfunction and endoplasmic reticulum stress). This localised cycle is consolidated and amplified by higher-level regulatory factors—including developmental epigenetic programming, the gut-brain-ovary axis, and circadian rhythms—ultimately establishing a pathologically entrenched state. Finally, this network-based understanding informs future research pathways, focusing on how emerging strategies—such as multi-omics integration, artificial intelligence, and stem cell secretome studies—can advance phenotype-directed precision interventions. This review aims to provide a systematic, dynamic theoretical framework for understanding the complex pathogenesis of PMOS and its potential therapeutic targets.

## 1 Introduction

Polyendocrine metabolic ovarian syndrome (PMOS), formerly known as polycystic ovary syndrome (PCOS), is a common and heterogeneous endocrine-metabolic disorder characterized by reproductive, endocrine, metabolic, and ovarian abnormalities. In May 2026, following a multistep global consensus process involving individuals with lived experience, healthcare professionals, researchers, and international professional organizations, the condition was renamed from PCOS to PMOS (1). The updated terminology was proposed to better reflect the multisystem nature of the disorder and to reduce the longstanding misconception that ovarian “cysts” represent its defining pathological feature. Importantly, this nomenclature change does not represent the recognition of a new disease entity or a fundamental alteration of the existing evidence-based diagnostic and management framework. In the present review, PMOS is therefore used as the current terminology when discussing the disorder in a contemporary context, whereas PCOS is retained when referring to historical diagnostic criteria, previously published studies, or terminology used in the original literature.

PMOS is a common endocrine and metabolic disorder among women of reproductive age, with a global prevalence ranging from 6% to 20% (2, 3). As a highly heterogeneous syndrome, it presents with diverse clinical phenotypes. According to the International Evidence-Based Guidelines for the Assessment and Management of PMOS (4), the diagnosis of adult PMOS requires fulfilment of at least two of the following three criteria: ① ovulatory dysfunction with menstrual cycle irregularities or amenorrhoea; ② clinical and/or biochemical manifestations of hyperandrogenism; ③ polycystic ovarian morphology and/or enlarged ovaries and/or elevated anti-Müllerian hormone (AMH) levels. PMOS not only represents the primary cause of anovulatory infertility (5), significantly impacting female reproductive health, but also carries markedly elevated risks of long-term complications including insulin resistance, obesity, type 2 diabetes, cardiovascular disease, and endometrial cancer (6–8), posing substantial challenges to women’s health throughout their life cycle. Consequently, a thorough analysis of its core pathophysiological mechanisms is crucial for developing effective intervention strategies.

Within the complex pathological network of PMOS, impaired follicular development is considered the central component of reproductive dysfunction (9). Under normal physiological conditions, follicular development undergoes recruitment, selection, maturation, and ultimately ovulation, maintained by a dynamic equilibrium precisely regulated by gonadotropins. However, in PMOS patients, follicular development is markedly disrupted. A hallmark pathological feature is the accumulation of numerous small antral follicles (diameter 2–9 mm) within the ovaries, which fail to progress into dominant follicles capable of ovulation, presenting a state of developmental arrest (10, 11). This transition from an ‘ordered dynamic process’ to ‘disordered static retention’ not only directly causes ovulatory dysfunction and infertility, but the underlying molecular and cellular dysregulation also represents a critical scientific question requiring urgent elucidation in current PMOS research.

Over the past decades, substantial progress has been made in elucidating the mechanisms underlying follicular development disorders in PMOS. Insulin resistance and compensatory hyperinsulinaemia, hallmark features of PMOS, are recognised as major contributors to abnormal follicular development. These effects may be mediated through several pathways supported by clinical and preclinical evidence, including stimulation of ovarian androgen production, amplification of LH effects, and disruption of hormonal signalling pathways (12–14). Dysfunction within the hypothalamic-pituitary-ovarian (HPO) axis is closely associated with, and may further contribute to, the ovarian hyperandrogenic environment and ovulatory dysfunction (15, 16). However, a single-mechanism perspective struggles to account for the complexity and heterogeneity of follicular arrest. Recent studies suggest follicular development relies on a finely tuned microenvironmental network comprising granulosa cells (GCs), oocytes, immune cells, and somatic cells, whose functional coupling depends on continuous molecular and metabolic dialogue. In PMOS, neuroendocrine and metabolic dysregulation disrupts this local homeostasis, causing bidirectional communication breakdown. This triggers immune cell infiltration, polarisation imbalance, and organelle stress, forming a self-reinforcing local pathological feedback loop. This loop is further integrated and amplified by cross-system factors including developmental programming, circadian rhythms, and gut microbiota.

The impaired follicular development observed in PMOS does not stem from a single pathway defect, but rather represents the local manifestation within the ovaries of a “pathological ecosystem” formed by the dysregulation of multiple physiological systems interacting systemically and locally. This paper aims to transcend superficial descriptions, systematically elucidating how diverse networks—including metabolic, immune, and epigenetic pathways—integrate through key molecular signals and potentially contribute to a local microenvironment associated with impaired preantral follicular development and defective dominant follicle selection. This encompasses regulation from the hypothalamic-pituitary-ovarian axis down to the ovarian granulosa cell microenvironment. This provides a robust theoretical foundation and innovative insights for discovering novel biomarkers and developing targeted therapeutic strategies (See **Figure 1**).

**Figure 1:**
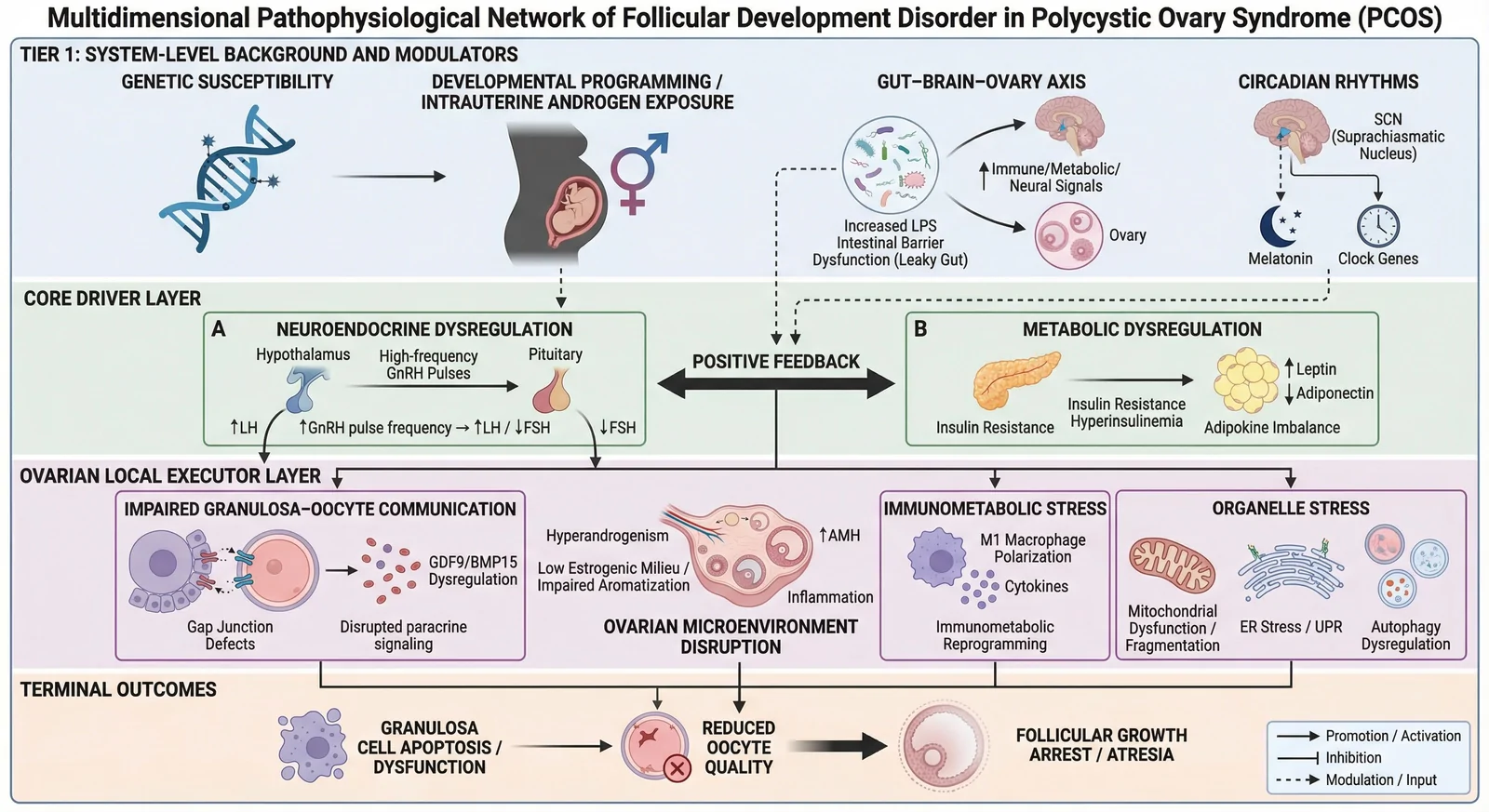
Multidimensional pathophysiological network underlying follicular developmental impairment in Polyendocrine metabolic ovarian syndrome (PMOS). Genetic susceptibility, developmental programming, the gut–brain–ovary axis, and circadian rhythms modulate neuroendocrine and metabolic dysregulation. These systemic abnormalities converge on the ovarian microenvironment, leading to hyperandrogenism, impaired granulosa–oocyte communication, immunometabolic activation, mitochondrial and endoplasmic reticulum (ER) stress, and autophagy dysregulation, ultimately resulting in granulosa-cell dysfunction, reduced oocyte competence, and follicular arrest/atresia. Solid arrows indicate activation or promotion, blunt-ended lines indicate inhibition, and dashed arrows indicate modulatory input.

## 2 Neuroendocrine and metabolic network dysregulation

Follicular development disorders are a near-universal core pathophysiological feature in PMOS patients (12, 13). This phenomenon stems not only from intrinsic ovarian dysfunction but also from widespread disruption in higher-level neuroendocrine regulation and systemic metabolic status (16, 17). These two systems form a vicious cycle of pathophysiological interplay through hormones, cytokines, and metabolic byproducts, driving follicular development off its normal trajectory (16).

### 2.1 GnRH pulse-induced LH/FSH imbalance

Normal follicular development relies upon negative feedback regulation of the hypothalamic-pituitary-ovarian axis (17). In PMOS, dysfunction of this axis constitutes its classic and core endocrine feature, rooted in rhythmic dysregulation (17–22).

GnRH neurons within the hypothalamic arcuate nucleus serve as the master switch of the reproductive axis, with their pulsatile secretion pattern governing pituitary gonadotropin release (18–21). Substantial evidence indicates that in PMOS patients and animal models, the frequency of the hypothalamic GnRH pulse generator is significantly accelerated (18). The aetiology of this high-frequency pulsing is multifactorial, involving pivotal dysregulation of KNDy neurons, remodelling of the neurotransmitter environment, and the effects of androgens and insulin (19–21). Pituitary gonadotroph cells exhibit a ‘preferential’ response to different GnRH pulse frequencies (18, 22). High-frequency GnRH pulses preferentially drive luteinising hormone (LH) secretion, while follicle-stimulating hormone (FSH) secretion is relatively insufficient. This results in characteristic hyper-LH ocaemia and an elevated LH/FSH ratio (22).

Persistently elevated LH levels excessively stimulate the proliferation and hyperfunction of theca cells, enhancing the activity of key androgen synthesis enzymes such as CYP17A1. This leads to the overproduction of androstenedione and testosterone, constituting the direct cause of ovarian-derived hyperandrogenism (23, 24). Concurrently, the relative insufficiency of FSH diminishes its capacity to drive antral follicle growth, promote granulosa cell proliferation, and activate aromatase (25). Consequently, follicles struggle to establish the normal ‘low androgen-high oestrogen’ dominant environment (23–25).

A deeper impact arises from the abnormal microenvironment induced by local hyperandrogenism and hyperinsulinemia, which causes premature and abnormally high expression of LH receptors in the granulosa cells of the antral follicles (26–29). At this stage, the abnormally elevated LH acts on these immature LH receptors, inducing premature luteinisation of the granulosa cells (26). Consequently, the follicular pool fails to receive sufficient selection and growth signals, ultimately leading to numerous follicles developing simultaneously yet stalling concurrently (25, 26). This manifests as the characteristic polycystic appearance on ultrasound. This represents one of the critical junctures in follicular arrest.

However, an elevated LH/FSH ratio is not a prerequisite for PMOS (30). In some lean PMOS women, LH levels may remain within the normal range. This suggests that abnormal GnRH pulsatility may exert effects through alternative downstream pathways, or that distinct endotypic subgroups exist (30). Consequently, it is more accurate to regard LH/FSH imbalance as a key pathophysiological amplifier rather than a mere initiating factor (18–22, 30). Its core mechanism lies in synergising with metabolic dysregulation to jointly establish a follicular microenvironment characterised by hyperandrogenism and disrupted developmental timing (23–25).

### 2.2 Insulin resistance and hyperinsulinaemia

Insulin resistance and compensatory hyperinsulinaemia constitute the most prevalent metabolic features of PMOS, observed in the vast majority of its phenotypes (12–14, 27–29). The ovary serves as a critical peripheral target organ for insulin, acting as a receptor for systemic metabolic disturbance signals (27–29). Insulin directly cross-reacts with the insulin-like growth factor-1 (IGF-1) receptor on theca cells or activates the ovarian CYP17A1 key enzyme for androgen synthesis via its post-receptor PI3K/Akt and MAPK signalling pathways (27–29). Concurrently, hyperinsulinaemia directly suppresses hepatic synthesis of sex hormone-binding globulin (SHBG) (31), elevating circulating free, bioactive testosterone levels and significantly exacerbating clinical hyperandrogenism (27–29, 31). At the granulosa cell level, insulin resistance reduces responsiveness to follicle-stimulating hormone (FSH) downstream of the insulin receptor substrate (IRS)-PI3K/AKT signalling pathway (32–34). This diminishes FSH’s biological effects, impairing its ability to effectively induce aromatase expression. Consequently, the conversion of androgens to oestrogens is inhibited, exacerbating the imbalance of ‘elevated androgens and reduced oestrogens’ within the follicle (32–34).

Thus, metabolic dysregulation and reproductive endocrine abnormalities form a mutually reinforcing positive feedback loop (12–14, 23, 24, 27–29). Persistent hyperinsulinaemia and insulin resistance constitute the metabolic dysfunctional foundation within the follicular microenvironment (27–29, 31–34). This state not only directly disrupts the normal function of follicular granulosa cells but also markedly reduces follicular sensitivity to key developmental signals, thereby undermining their normal growth and differentiation capacity (24, 27–29, 31–34).

### 2.3 Adipose tissue: an active endocrine and inflammatory organ

Adipose tissue, particularly visceral fat, is no longer regarded as a passive energy reservoir in PMOS (35, 36). It has been redefined as a functionally hyperactive endocrine and immune organ, whose secretion of multiple adipokines profoundly influences the pathological progression of PMOS (37–40).

Hyperleptinaemia and leptin resistance are frequently observed in PMOS patients (35, 36). Leptin normally acts on the hypothalamus to regulate GnRH secretion; however, its resistant state disrupts normal HPO axis control, exacerbating abnormal GnRH pulsatility through central mechanisms (35, 36). At the ovarian level, studies confirm leptin directly inhibits granulosa cell proliferation and oestrogen synthesis while stimulating theca cells to produce androgens (37). Concurrently, adiponectin—which possesses insulin-sensitising and anti-inflammatory effects—is markedly reduced in PMOS patients, with diminished adiponectin receptors in theca cells (38–40). This not only diminishes systemic insulin sensitivity but may also impair its direct effects on ovarian function, thereby losing its protective regulatory role in follicular development (38–40).

PMOS frequently coexists with dyslipidaemia. Excessive circulating free fatty acids (FFAs) exert lipotoxicity on non-adipose tissues (41, 42). Within the ovary, FFAs directly damage granulosa cells and oocytes by inducing endoplasmic reticulum stress, oxidative stress, and mitochondrial dysfunction through excessive reactive oxygen species production. This ultimately leads to apoptosis and diminished oocyte quality, resulting in follicular atresia and impaired fertility (41, 42).

In summary, dysfunction of the HPO axis, in conjunction with systemic metabolic syndrome, constitutes the core pathological driver of the disease (12–17, 27–29). By collectively establishing an endocrine environment characterised by hyperandrogenism, hyperinsulinemia, and chronic low-grade inflammation, it persistently stimulates and disrupts ovarian function regulation, resulting in the widespread occurrence of follicular development disorders in PMOS patients (23, 24, 43–46). However, these factors alone cannot fully explain the stable and difficult-to-reverse state of follicular arrest (32–34). Recent research suggests that persistent endocrine dysregulation disrupts intra-follicular communication systems and triggers immune stress responses within cellular organelles. Consequently, dysfunction of the HPO axis and insulin resistance are recognised as drivers initiating disease onset and accelerating pathological progression. Yet the intrinsic defect underpinning the core pathological phenotype of follicular arrest lies in the disruption of cellular communication networks within the follicular microenvironment and the ensuing vicious cycle of intracellular stress responses (41–46). Consequently, the following discussion will focus on the follicle as a functional unit, systematically analysing the mechanisms of intercellular communication disruption and the formation and maintenance of local stress feedback loops. This will enable the comprehensive construction of a multi-layered pathological network underlying follicular arrest in PMOS.

## 3 Intrafollicular cellular dialogue and dysfunction

In PMOS, follicular arrest manifests not only as morphological stagnation; its underlying pathophysiological mechanism involves the remodelling of the follicular functional unit (47–52). The collaboration between granulosa cells and oocytes relies on a multi-layered, intricate signalling network, primarily comprising endocrine signals, paracrine/autocrine signals, and direct intercellular communication (47–49, 53–55). Current perspectives suggest that within the PMOS pathological state, widespread dysregulation of these signalling systems collectively leads to the failure of bidirectional communication between granulosa cells and oocytes (49–52). This prevents the attainment of critical signalling thresholds required for follicular maturation, ultimately causing follicles to arrest at the early antral stage (47, 48, 51, 52).

### 3.1 Endocrine hormone signalling dysregulation

During the physiological cycle, follicle-stimulating hormone (FSH) secreted by the pituitary gland serves as the primary driver of follicular development (53). FSH binds to the follicular cell-surface FSH receptor (FSHR), initiating a cascade of events via the cAMP-PKA signalling pathway. This includes cellular proliferation, induction of aromatase expression, and LH receptor expression (54, 55). The pathological consequence is not merely insufficient oestrogen synthesis, but the establishment of a ‘hyperandrogenic-hypoestrogenic’ endocrine homeostasis (53–55).

CYP19A1 (aromatase) is the rate-limiting enzyme for the conversion of androstenedione and testosterone to oestrone and E2 in GCs, and is finely regulated by FSH (58). In GCs from PMOS patients, downregulated aromatase expression or insufficient activity is prevalent, involving mechanisms spanning multiple levels such as insulin resistance and hyperinsulinaemia, hyperandrogenism, inflammatory factors, and epigenetic regulation (32, 58–63). Insulin synergises with FSH to promote aromatase expression (59). However, under insulin-resistant conditions, reduced responsiveness to FSH downstream of the IRS-PI3K/AKT signalling pathway diminishes FSH’s biological effects, thereby failing to effectively induce aromatase expression (32). Excessively high androgen levels within GCs can directly or indirectly suppress aromatase transcription and activity via androgen receptor (AR) signalling, or by recruiting epigenetic modification complexes such as histone deacetylases (HDACs), thereby maintaining the gene promoter region in a silenced chromatin state (60, 61). Concurrently, chronic low-grade inflammatory factors within the PMOS ovarian microenvironment suppress aromatase activity (62); epigenetic mechanisms including DNA methylation and histone modifications (63) have also been demonstrated to contribute to CYP19A1 gene silencing.

Oestrogen functions not only as a feedback-regulating hormone but also as a crucial paracrine/autocrine factor locally driving granulosa cell proliferation within the follicle (53–55, 58). It promotes FSH and LH receptor expression, sustains follicular viability, and inhibits apoptosis (54, 55). Insufficient oestrogen levels result in diminished granulosa cell proliferation, preventing follicles from attaining the volume required for dominance. This leads to delayed or abnormal LH receptor acquisition and reduced resistance to apoptosis, predisposing follicles to premature atresia (53–55).

At the microenvironmental level, persistent accumulation of androgens within the follicular fluid further activates the androgen receptor (AR) signalling pathway and exacerbates oxidative/endoplasmic reticulum stress. This, in turn, feedback-inhibits aromatase function, establishing a vicious cycle of ‘androgen accumulation → aromatase inhibition → further androgen accumulation’ (60). This closed loop traps the follicular microenvironment in a pathological steady state that inhibits dominant follicle progression, characterised by hyperandrogenism, hypoestrogenism, and chronic low-grade inflammation (60–63).

Current conclusions regarding aromatase expression or activity in PMOS remain inconsistent. Some studies suggest reduced levels, while others report no significant abnormalities. These discrepancies may stem from sample heterogeneity, inadequate patient stratification, and limitations in detection methods. Future research thus requires refined stratification designs to provide more reliable functional indicators for accurately assessing individual follicular developmental potential.

### 3.2 Abnormal anti-Müllerian hormone signalling

Anti-Müllerian hormone (AMH) and its downstream signalling pathways exhibit dual roles in the pathophysiology of PMOS. On the one hand, elevated serum levels serve as a crucial clinical indicator for diagnosing PMOS and assessing ovarian reserve. On the other hand, excessively high AMH and its overactivated signalling transform it from a regulator that physiologically inhibits excessive recruitment of primordial follicles into a key inhibitory factor that pathologically impedes the selection of dominant follicles from the follicular pool (64, 65).

During the normal ovarian cycle, AMH secreted by theca cells of small antral follicles primarily acts paracrinally to reduce the sensitivity of surrounding follicles to FSH, preventing excessive recruitment of antral follicles and ensuring the recruitment of dominant follicles. However, in PMOS patients, markedly elevated serum and follicular fluid AMH levels bind to its type II receptor, leading to excessive phosphorylation and sustained activation of the downstream SMAD signalling pathway (65, 66). This pathological signalling significantly suppresses CYP19A1 expression and activity (65, 66). Acting synergistically with factors such as hyperandrogenism and insulin resistance, it further exacerbates the local ‘low oestrogen’ environment within follicles. This pathway also reduces FSH receptor responsiveness, elevates the FSH signalling threshold, and inhibits the synchronisation and selection processes within the follicular pool (64–66).

Elevated circulating and follicular AMH levels and altered AMH expression in granulosa cells have been consistently observed in women with PMOS, providing direct human evidence for dysregulated AMH signalling (64–67). However, the extent to which excessive AMH signalling directly causes follicular arrest, and the molecular pathways mediating this effect, has been established largely through *in vitro* granulosa-cell experiments and animal models; thus, AMH should presently be regarded as an important contributor rather than a definitively established primary driver of follicular arrest (67).

### 3.3 Impaired gap junction communication

During follicular development, gap-junction channels formed by connexins serve as a core structure maintaining synchronised material exchange and signalling between granulosa/cumulus cells and oocytes. This pathway directly mediates the bidirectional transport of small metabolic molecules (e.g., glucose and amino acids), second messengers, and ions, and is crucial for maintaining oocyte meiotic arrest, energy homeostasis, and antioxidant defence (47, 48).

Under PMOS pathological conditions, this critical communication system appears to be disrupted (56, 57). Studies using patient-derived GCs have reported altered expression of connexin 37 (Cx37) and connexin 43 (Cx43) in PMOS (56), providing human evidence of abnormalities in gap-junction-associated molecular profiles. *In vitro* studies further indicate that both dihydrotestosterone and a hyperinsulinaemic environment can downregulate Cx43 expression in GCs and disrupt its membrane localisation, leading to impaired gap-junction function. Experimental studies suggest that loss of gap-junction function can disrupt metabolic coupling between oocytes and surrounding granulosa cells, thereby limiting metabolic and signalling support to the oocyte. Such disturbances may contribute to reduced ATP availability, redox imbalance, and instability of cAMP signalling required for maintaining meiotic arrest, consequently compromising oocyte maturation and developmental competence (47, 57).

From a conceptual perspective, severe disruption of metabolic and signalling support to the oocyte could contribute to developmental arrest of the follicular unit. This has been proposed as a potential biological ‘stop-loss’ response that may limit continued resource investment in a follicle with reduced developmental competence. Thus, disruption of gap-junction communication may contribute to reduced oocyte developmental competence and may represent one mechanism associated with follicular arrest and atresia (47, 56, 57). However, most available human data are derived from granulosa–oocyte complexes obtained during assisted reproductive cycles, which may already have been influenced by exogenous gonadotropin stimulation and oocyte retrieval procedures (56, 57). Therefore, although altered connexin expression has been observed in patient-derived samples, direct evidence that gap-junction dysfunction drives follicular arrest under natural-cycle PMOS conditions remains limited, and much of the mechanistic evidence currently derives from cellular or experimental models (56, 57).

### 3.4 Communication impairment within the transforming growth factor-β family

The TGF-β superfamily, encompassing activins, inhibins, BMPs, and GDFs, constitutes a central paracrine pathway regulating oocyte function via surrounding granulosa cells (49, 50). Dysfunction within this signalling pathway reflects the failure of coordination mechanisms between oocytes and granulosa cells within the follicular microenvironment, potentially triggering a cascade of pathological responses (49–51).

Relevant studies indicate (51) that altered expression profiles of growth differentiation factor 9 (GDF9) and bone morphogenetic protein 15 (BMP15) weaken oocyte regulation of granulosa cells (GCs). This further exacerbates abnormal steroid production within GCs, disrupting their metabolic reprogramming (51). The resulting harmful metabolic by-products directly attack oocytes via paracrine pathways, inducing mitochondrial damage and DNA oxidation (52).

These alterations form a vicious cycle: oocytes deteriorate in quality due to metabolic toxicity, their secreted GDF9/BMP15 signalling becomes further disrupted, exacerbating granulosa cell dysfunction, which in turn generates more toxic products that reciprocally damage the oocytes (51, 52). This traps oocytes and granulosa cells in a pathologically self-damaging state, fundamentally obstructing the achievement of follicular dominance(See **Figure 2**).

**Figure 2:**
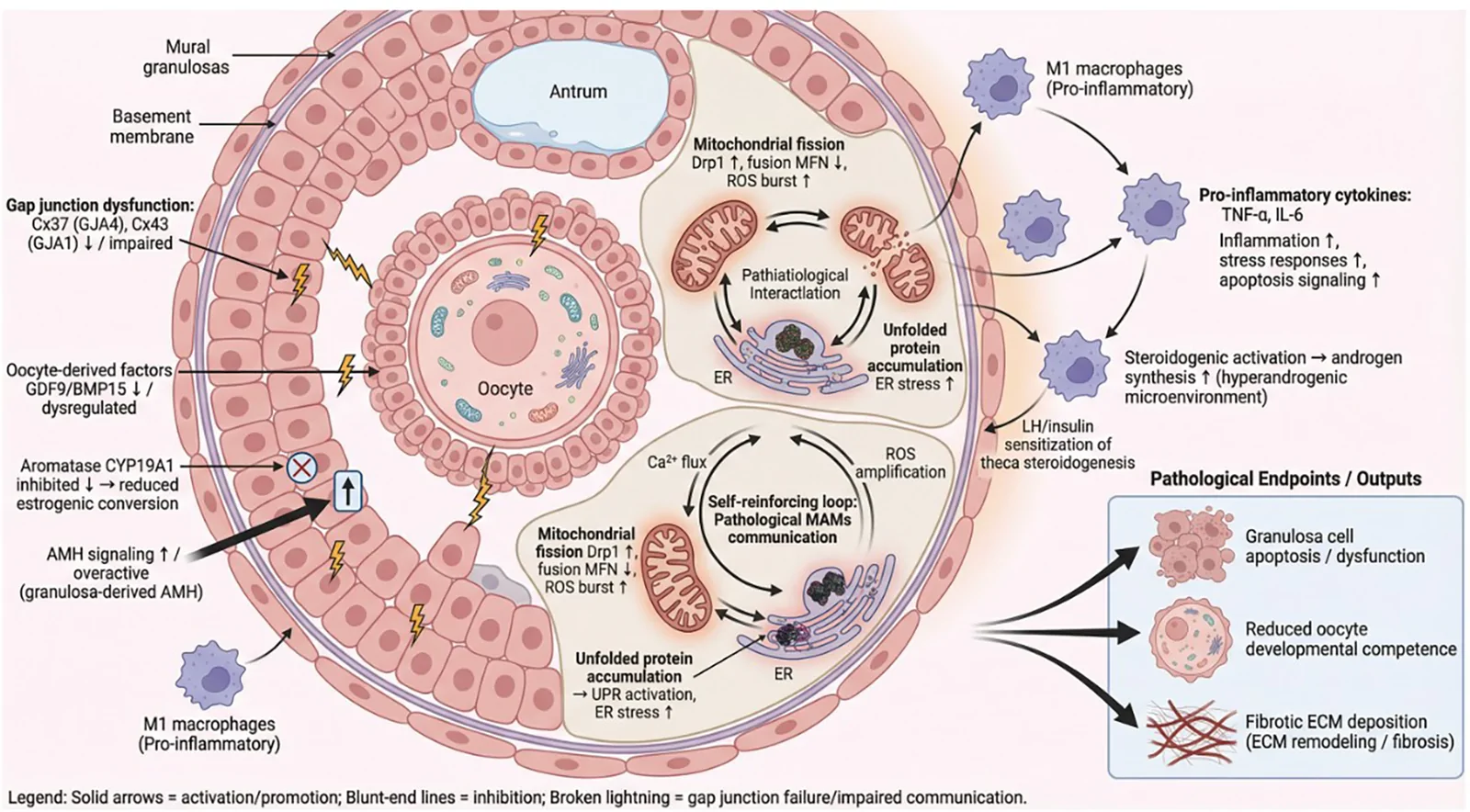
Dysfunctional intercellular signalling within the follicular microenvironment in PMOS, highlighting disrupted communication between granulosa cells and oocytes via gap junctions and TGF-β family pathways. Impaired gap-junction communication and dysregulated growth differentiation factor 9 (GDF9)/bone morphogenetic protein 15 (BMP15) signalling disrupt granulosa–oocyte interactions. These abnormalities interact with mitochondrial dysfunction, endoplasmic reticulum (ER) stress, reactive oxygen species (ROS) accumulation, and pro-inflammatory macrophage activation, promoting granulosa-cell apoptosis, reduced oocyte developmental competence, and extracellular matrix (ECM) remodelling. Solid arrows indicate activation/promotion, blunt-ended lines indicate inhibition, and broken lightning symbols indicate impaired intercellular communication.

## 4 Immune infiltration - metabolic dysregulation - interplay of organelle stress

Under the sustained influence of metabolic and neuroendocrine dysregulation, the local follicular microenvironment within the ovary represents an important site at which these systemic abnormalities converge (43–46). These disturbances may trigger a cascade of progressively intensifying cellular stress responses, potentially forming a pathological closed loop within the follicle (68–70). Such processes may contribute to the transition from initially reversible functional dysfunction to a more persistent state of developmental arrest (71–74). Importantly, the evidence supporting individual components of this proposed closed loop is heterogeneous: some alterations have been observed directly in human PMOS ovarian tissues, follicular fluid, or patient-derived cells, whereas many of the causal links between these alterations and follicular arrest have been established predominantly in cellular and animal models (43–45, 70, 75).

The proposed pathological logic of this closed loop can be summarised as follows: Immune cell infiltration and chronic low-grade inflammatory mediators (43–46)→ metabolic reprogramming and functional disruption (68, 69, 77) → mitochondrial dysfunction and increased reactive oxygen species generation (69, 70, 78–85) → organelle stress, including endoplasmic reticulum stress and calcium homeostasis imbalance (79, 82, 86–91) → increased susceptibility to apoptosis, local fibrosis, and abnormal angiogenesis (73, 74, 86, 88) → further exacerbation of the inflammatory environment, hyperandrogenism, and insulin resistance (44, 76, 77) → progressive impairment of follicular development and potentially a stable arrest state. This sequence should be interpreted as an integrative mechanistic framework rather than as a fully established causal cascade in human PMOS (43–45, 70, 75).

### 4.1 Chronic low-grade inflammation

Increased immune cell infiltration has been reported in the ovarian stroma and follicular fluid of PMOS patients, accompanied by persistently elevated levels of pro-inflammatory factors (43–45). These observations provide direct human evidence for local inflammatory and immune dysregulation in PMOS, although the mechanisms responsible for its initiation and maintenance have been investigated extensively in experimental models (43–46). Experimental and translational studies suggest that several factors may contribute to this process: hyperinsulinaemia and hyperandrogenism can promote immune-cell activation; lipogenic inflammatory mediators from dysfunctional adipose tissue may reach the ovaries via the circulation; additionally, metabolic endotoxinaemia associated with gut microbiota dysbiosis may influence the ovarian microenvironment through systemic circulation (46). Consequently, local ovarian anti-inflammatory and repair mechanisms may become impaired, favouring a state of chronic pro-inflammatory factor overload (43–46).

Persistent low-grade inflammation may impair follicular development (43–46, 76). Experimental studies indicate that inflammatory mediators such as TNF-α can exacerbate ovarian insulin resistance by inducing serine phosphorylation of insulin receptor substrate (IRS); concurrently, TNF-α may upregulate key enzymes for androgen synthesis in theca cells and inhibit aromatase activity in granulosa cells, thereby promoting a pathological ‘hyperandrogenic-hypooestrogenic’ state (76). Persistent inflammatory stress may also interfere with the temporal window required for follicular dominance selection, thereby contributing to impaired dominant follicle growth and selection (43–46, 76).

### 4.2 Immune cell infiltration

Within the ovarian immune microenvironment, macrophages play a pivotal role as active regulators of microenvironmental homeostasis. As crucial modulators, they directly participate in normal follicular development and ovulation, while their functional phenotype determines whether the local environment favours tissue repair or persistent injury (43, 92, 93). Human ovarian studies support altered macrophage abundance and/or macrophage-associated inflammatory signatures in PMOS; however, the functional consequences of specific macrophage phenotypes and their causal contribution to follicular arrest have been characterised more extensively in animal and cellular models (43, 77, 92, 93).

Macrophages are functionally categorised into pro-inflammatory M1 and anti-inflammatory/reparative M2 types (93). Under physiological conditions, these maintain a dynamic equilibrium, with M2 macrophages supporting follicular development, post-ovulatory angiogenesis, and tissue repair through secretion of factors such as transforming growth factor-β (TGF-β) (94, 95). Experimental studies suggest that under hyperinsulinaemic and hyperandrogenic conditions relevant to PMOS, macrophage polarisation may shift towards an M1-like pro-inflammatory phenotype (44). This phenotype can promote the release of pro-inflammatory factors such as TNF-α and IL-6, which may disrupt the normal function of granulosa and theca cells, inhibit aromatase activity, and exacerbate local ovarian androgen accumulation (44, 77). Consequently, the selection and growth of dominant follicles may be impaired (77).

This dysregulated macrophage polarisation may promote a persistently pro-inflammatory, pro-fibrotic, and hyperandrogenic ovarian microenvironment (44, 77). Consequently, it may further exacerbate local metabolic and endocrine dysregulation, disrupting the microenvironment essential for follicular dominance (44, 77). These alterations may increase the susceptibility of follicles to developmental arrest at the antral stage and subsequent atresia. Thus, although human studies support the presence of ovarian immune dysregulation in PMOS, direct evidence that altered macrophage polarisation itself drives follicular arrest in humans remains limited, with much of the mechanistic evidence derived from experimental models (43, 44, 77, 92, 93).

### 4.3 Immunometabolic reprogramming

Recent research suggests that the metabolic states of immune cells and somatic cells not only reflect their functional states but also serve as key factors actively regulating cellular phenotypes (68, 69). Within the PMOS follicular microenvironment, a bidirectionally reinforcing pathological coupling may emerge between the immunometabolic reprogramming of immune cells and granulosa cells and the chronic inflammatory state. This process may represent an important mechanism contributing to amplification of the local pathological feedback loop within the ovary (68, 69).

Metabolic reprogramming can influence cellular functional phenotypes (69). Macrophage polarisation is intrinsically linked to their metabolic profile (68). Similarly, the steroid synthesis capacity, proliferative activity, and stress adaptation capabilities of granulosa cells are highly dependent on the efficiency of mitochondrial oxidative phosphorylation (69, 70, 96). Within the PMOS pathological environment, experimental studies suggest that hyperinsulinaemia and inflammatory cytokines can shift cellular metabolism towards an aerobic glycolysis-dominant pattern (68). Whilst this metabolic shift enables rapid ATP production and biosynthetic precursor generation, it may disrupt intracellular redox balance, generating excessive reactive oxygen species (ROS) and inducing oxidative stress (68, 69). Excessive oxidative stress can damage DNA, proteins, and lipids, and may promote endoplasmic reticulum stress and mitochondrial dysfunction (69). Consequently, inflammation and metabolic disorders do not exist in isolation but may form interconnected regulatory networks through shared metabolic intermediates and signalling pathways. This interdependence means that intervention targeting one pathway may be limited in effect due to compensatory activation of another pathway (68, 69).

### 4.4 Mitochondrial dynamics and dysfunction

Mitochondria serve as the cellular hub for energy metabolism and signal integration, while also regulating apoptosis, calcium homeostasis, and reactive oxygen species production (96). They not only provide stable, sustained energy for rapidly proliferating granulosa cells and oocytes undergoing meiotic resumption, but also ensure steroid hormone synthesis in granulosa cells (70). In PMOS, mitochondrial structural and functional abnormalities have been reported in patient-derived granulosa cells, providing direct human evidence that mitochondrial dysfunction is associated with the disease phenotype. However, the detailed causal sequence linking mitochondrial network disruption to follicular arrest has been characterised predominantly in cellular and animal studies (70, 75).

Mitochondria achieve adaptive regulation of their morphology, distribution, and function through continuous processes of fission and fusion. This is crucial for inter-mitochondrial exchange, damage repair, and functional coordination (78, 79). Studies using PMOS-derived cells and experimental models have reported abnormalities in mitochondrial dynamics, with increased expression and activity of the fission protein Drp1, while key fusion-mediating proteins (such as MFN1, MFN2, and OPA1) have been reported to show reduced expression (78, 79). Concurrently, electron microscopy and confocal imaging studies have demonstrated mitochondrial morphological abnormalities, including an increased prevalence of short rod-like or granular mitochondria and loss of the normal elongated, interconnected network structure (78, 79). Experimental evidence further suggests that these alterations may be associated with two hallmark pathological states of PMOS: hyperandrogenism and insulin resistance (79, 80).

Evidence from cellular and animal models suggests that the multifaceted functional disruption caused by mitochondrial dyskinesis may impair follicular development through multiple pathways, including energy metabolism, oxidative stress, and calcium homeostasis (81, 82). Fragmented mitochondria exhibit reduced electron transport chain (ETC) efficiency, diminishing oxidative phosphorylation capacity and potentially reducing cellular ATP availability. Reduced ATP production and abnormalities in mitochondrial function have also been observed in patient-derived granulosa cells, supporting the clinical relevance of this phenotype; however, its direct contribution to oocyte developmental arrest remains less clearly established in humans (70, 75, 83, 84). This energy deficit may impair the rapid proliferation of granulosa cells and the biosynthetic pathways required for steroid hormone synthesis. Furthermore, it may compromise the ability to meet the high energy demands necessary for oocyte maturation, thereby contributing to impaired developmental competence (83, 84).

Concurrent ETC dysfunction can promote excessive ROS production, which subsequently damages lipids, proteins, and DNA (81, 85). Experimental studies indicate that excessive mitochondrial oxidative stress can compromise follicular membrane integrity, oocyte spindle stability, chromosomal structure, and telomere maintenance, and may promote granulosa cell apoptosis (81, 85). These abnormalities may further favour localised androgen accumulation within follicles and worsen insulin resistance (81, 85). The decline in mitochondrial membrane potential may compromise calcium buffering capacity, elevating cytoplasmic calcium concentrations (82). This can further impair mitochondrial function and enhance ROS generation, while potentially activating calcium-dependent proteases and apoptotic pathways (82, 86). These effects collectively may contribute to increased granulosa cell apoptosis, disrupted steroid synthesis, and diminished oocyte quality (81, 82, 85, 86). Together, these observations support mitochondrial dysfunction as an important component of the pathological follicular microenvironment; nevertheless, whether it acts as an independent or primary driver of follicular arrest in human PMOS remains to be established (70, 75, 81–86).

### 4.5 Endoplasmic reticulum stress

The endoplasmic reticulum serves as the primary site for protein synthesis, folding, and steroid hormone production (87–89). Studies using patient-derived granulosa cells and experimental models suggest that prolonged exposure of PMOS granulosa cells to metabolic and oxidative stress environments may promote abnormal accumulation of unfolded or misfolded proteins within the ER lumen. This triggers endoplasmic reticulum stress (ERS) and activates the unfolded protein response (UPR) (87). The UPR aims to restore cellular homeostasis by alleviating ER load. However, persistent ER stress signalling may promote granulosa cell apoptosis via pathways such as CHOP (88) and disrupt steroid hormone synthesis (89), thereby contributing to local microenvironmental disorder within PMOS (87–89).

The endoplasmic reticulum and mitochondria are structurally and functionally tightly coupled via the mitochondria-associated endoplasmic reticulum membrane (MAMs) (79). Experimental evidence suggests that under PMOS-related pathological conditions, their physiological dialogue may shift towards maladaptive interaction (79, 90, 91). Endoplasmic reticulum stress can promote abnormal calcium ion release into mitochondria, exacerbating calcium overload and dysfunction; conversely, excessive reactive oxygen species (ROS) generated by mitochondria can diffuse into the endoplasmic reticulum, further intensifying protein misfolding and endoplasmic reticulum stress (79, 90, 91). This mutual amplification of stress signals may markedly diminish the cell’s capacity to maintain homeostasis (79, 90, 91).

### 4.6 Dysregulation of autophagy

Autophagy constitutes a vital cellular process whereby damaged components are degraded via lysosomes to maintain homeostasis (71, 72). Within the stressed environment of PMOS, the dynamic equilibrium of autophagy is disrupted, with existing research frequently reporting contradictory findings of both autophagy activation and functional defects (71, 72, 97–100). This reflects autophagy’s complex dual role in PMOS pathogenesis.

During the early stress phase of PMOS, autophagy may be moderately activated to clear dysfunctional mitochondria and misfolded protein aggregates. When stress signals become excessive, the autophagic process may become impaired, leading to autophagic flow blockage and ineffective clearance of damaged mitochondria and protein aggregates. At this juncture, autophagy transitions from a protective mechanism to a state of dysregulation, leading to intracellular accumulation of harmful substances (71, 72).

Discrepancies in the literature may stem from multiple factors, including subject heterogeneity and limitations in detection methods (97–99). Consequently, future research should employ techniques for longitudinal monitoring of autophagy flux dynamics and conduct differentiated assessments across distinct PMOS subtypes (97, 99). These observations suggest that therapeutic strategies targeting autophagy may need to be adapted to the specific pattern of autophagic dysfunction: for subtypes with insufficient autophagy function, promoting autophagy activation may be beneficial; whereas for subtypes exhibiting autophagy flow blockage, restoring lysosomal clearance function may be more appropriate than merely enhancing autophagy initiation (97, 98).

### 4.7 Extracellular matrix remodelling and fibrosis

Experimental studies indicate that persistent stimulation of ovarian stroma by inflammatory cytokines and transforming growth factor-β (TGF-β) can promote fibroblast-to-myofibroblast transdifferentiation, leading to excessive production of extracellular matrix (ECM) components such as collagen. Concurrently, the dynamic equilibrium between matrix metalloproteinases (MMPs) and their tissue inhibitors (TIMPs) may be disrupted, resulting in inadequate degradation and abnormal deposition of the ECM (73, 74). Histological and molecular studies of human PMOS ovarian tissues have reported stromal and ECM abnormalities, supporting the clinical relevance of fibrosis-related remodelling; however, the specific molecular pathways driving progressive fibrosis and its direct causal contribution to follicular arrest have been established more extensively in cellular and animal models (73, 74).

Increased collagen deposition, tissue thickening, and stiffness within the ovarian capsule and follicular stroma may reduce the mechanical compliance required for normal follicular rupture during ovulation. These structural abnormalities could therefore contribute to ovulatory dysfunction; however, a direct causal relationship between ovarian fibrosis and luteinised unruptured follicle syndrome (LUFS) in human PMOS has not been firmly established. Concurrently, the abnormal ECM and fibrotic network may disrupt physiological angiogenesis, potentially impairing the efficient delivery of oxygen, nutrients, and hormonal signals essential for follicular growth and development (73, 74).

When fibrosis and ECM remodelling reach a certain threshold, experimental evidence suggests that the follicular microenvironment may transition from a dynamic, plastic functional unit into a pathologically constrained state with limited biological activity. At this stage, even if systemic metabolic and endocrine dysregulation is partially corrected, local structural abnormalities within the ovary may persist. This may partly explain why ovarian responsiveness and ovulatory function remain difficult to restore in some PMOS patients despite prolonged treatment (73, 74). Consequently, early intervention to prevent or delay irreversible structural alterations may hold significant clinical importance. Nevertheless, whether fibrosis itself constitutes a primary driver of persistent follicular arrest in human PMOS remains to be determined.

## 5 Epigenetics and developmental programming

PMOS exhibits marked familial clustering (30, 100). Recent integration of epigenetics with developmental origin hypotheses has provided novel temporal and molecular perspectives on its follicular development impairment (101, 102). This theory posits that embryonic exposure to hyperandrogenic environments constitutes the critical initiating factor for the subsequent adult PMOS phenotype in female offspring (101–103). Relevant animal model studies (101) demonstrated that maintaining intrauterine hyperandrogenism through testosterone injections consistently produced offspring exhibiting stable PMOS characteristics. Intrauterine androgen overload does not directly alter gene sequences. Instead, it disrupts the normal developmental trajectories of the foetal HPO axis, pancreatic β-cells, and adipose tissue, setting them onto an aberrant pattern (102, 104). Consequently, triggered by specific environmental factors around puberty, the PMOS phenotype ultimately manifests (102, 105).

Epigenetics constitutes a branch of genetics regulating gene expression through chemical modifications without altering DNA sequences. Such modifications—including DNA methylation, histone modifications, and non-coding RNAs—are established during development and can be relatively stably inherited, forming the core molecular mechanisms underpinning the developmental origin hypothesis (102, 106).

### 5.1 Abnormal DNA methylation

DNA methylation denotes the process whereby methyl groups supplied by S-adenosylmethionine are selectively added to the fifth carbon atom of the cytosine nucleotide in the cytosine-guanine dinucleotide, catalysed by DNA methyltransferases, forming 5-methylcytosine (107). Disrupted DNA methylation is pervasive in both granulosa cells and oocytes from PMOS ovaries, with these abnormalities directly causing follicular arrest by impairing critical signalling pathways. Researchers have discovered (108–110) that abnormal methylation of genes associated with androgen signalling pathways and insulin signalling pathways leads to hyperandrogenism and insulin resistance, subsequently affecting granulosa cell metabolism and function, thereby indirectly interfering with follicular development. Concurrently, studies suggest (111, 112) that methylation alterations in core follicular development regulators—such as anti-Müllerian hormone and follicle-stimulating hormone receptors—inhibit aromatase activity, reduce testosterone conversion to oestradiol, and suppress follicle-stimulating hormone’s promotion of dominant follicles, thereby impeding dominant follicular development.

### 5.2 Dysregulation of histone chemical modifications

Modifications such as histone acetylation and methylation collectively and precisely regulate chromatin accessibility and gene transcription (113, 114). Histone acetylation levels are jointly regulated by histone acetyltransferases (HATs) and histone deacetylases (HDACs) (114). Hyperandrogenism and insulin can influence the HATs/HDACs balance through multiple pathways. Hyperandrogenism may recruit HDACs to specific gene promoter regions via the androgen receptor (AR) signalling pathway, leading to localised hypoacetylation of histones and consequently suppressing a series of genes critical for meiotic and cytoplasmic maturation (61, 115).

### 5.3 Non-coding RNA regulatory networks

Non-coding RNAs (ncRNAs), primarily comprising microRNAs (miRNAs), long non-coding RNAs (lncRNAs), and circular RNAs (circRNAs), constitute a dynamic network that precisely regulates gene expression at both transcriptional and post-transcriptional levels (116). In PMOS, specific ncRNA expression profiles undergo significant alterations, playing a central role in integrating upstream endocrine, metabolic, and inflammatory signalling disturbances and translating these into downstream cellular dysfunctions (117, 118).

miRNA are short non-coding RNA sequences that negatively regulate gene expression at the transcriptional level. In PMOS patients, dysregulation of multiple key miRNAs has been demonstrated to profoundly involve follicular development disorders. Downregulation of miR-145 may abnormally activate pathways associated with granulosa cell proliferation by releasing inhibition of insulin receptor substrate 1 (IRS1); downregulated miR-92a/b expression correlates with disrupted androgen synthesis in theca cells; whereas elevated miR-200b/429 expression may modulate pituitary function (119–121). These miRNAs directly regulate steroidogenesis, insulin signalling, and cell cycle progression. lncRNAs exert more intricate regulatory functions through chromatin modifications, transcriptional interference, and ceRNA mechanisms (116, 122, 123). Alternatively, by recruiting chromatin-modifying complexes, they modulate gene cluster expression at the epigenetic and transcriptional levels, thereby influencing the balance of granulosa cell proliferation, apoptosis, and differentiation (122, 123).

ncRNAs, particularly those stably present in follicular fluid exosomes, are emerging as highly promising novel biomarkers and therapeutic intervention targets due to their excellent stability and tissue specificity (116, 124). They hold potential not only for PMOS disease classification but also, either intrinsically or through their specific antagonists, as novel tools for future precision interventions to disrupt specific pathological circuits.

## 6 Systemic regulatory mechanisms

Within the complex pathological network of PMOS, dysfunction of the HPO axis and systemic metabolic derangements represent major systemic abnormalities that may contribute to follicular dysfunction, whilst disrupted follicular communication and local stress responses may represent downstream mechanisms associated with developmental arrest. Additionally, two systems with systemic regulatory functions—the circadian rhythm system and the gut-brain-ovary axis—play pivotal regulatory and modifying roles (125, 134). While they may not constitute the primary aetiology of all PMOS cases, their dysregulation significantly exacerbates upstream signalling disturbances and alters the downstream ovarian microenvironment. Consequently, they may offer crucial mechanistic insights into the marked clinical heterogeneity observed in PMOS patients (125, 134).

### 6.1 Circadian rhythm dysregulation

The circadian rhythm system constitutes a self-sustaining network of multiple oscillators, comprising a central oscillator (the master biological clock) located in the suprachiasmatic nucleus (SCN) and secondary oscillators (peripheral biological clocks) within peripheral organs and tissues. By modulating circadian gene expression or protein levels, the body synchronises its internal clock with Earth’s environmental time, thereby generating approximately 24-hour rhythmic patterns in sleep-wake cycles, feeding behaviour, thermoregulation, and hormone secretion (126).

Recent studies (127, 128, 134) have revealed that peripheral organs (such as female reproductive tissues) express circadian genes, playing crucial roles in multiple reproductive processes including follicular development, ovulation, hormonal homeostasis coordination, embryo implantation, and hormone synthesis. Abnormalities in the light-dark cycle directly disrupt the SCN’s normal rhythmic output, weakening its inhibitory regulation of hypothalamic gonadotropin-releasing hormone (GnRH) neuron activity. This results in the loss of physiological rhythmicity in GnRH pulsatile secretion, leading to a tendency towards high-frequency secretion. Consequently, the imbalance in the LH/FSH ratio is exacerbated, causing the pituitary gland to transmit disordered growth regulatory signals to the ovaries (127, 129).

Substantial evidence indicates a close relationship between the biological clock and androgen production. Research by Chen, Johnson et al. (128, 130–132, 134) suggests clock genes participate in the transcription of key enzymes for steroid synthesis; BMAL1/CLOCK promotes aromatase expression, driving the conversion of androgens to oestrogens. Abnormal expression or function of circadian clock genes (such as CLOCK and BMAL1) reduces granulosa cell aromatase capacity while simultaneously increasing theca cell androgen synthesis. This creates the hyperandrogenic microenvironment characteristic of PMOS, impairing follicular quality and inhibiting follicular dominance (128, 130–132).

Concurrently, as a key hormone regulated by light-dark cycles, melatonin exhibits potent antioxidant and anti-inflammatory properties. Circadian rhythm disruption reduces nocturnal melatonin secretion peaks (133, 134), potentially weakening ovarian defences against oxidative stress and correlating with diminished ovarian reserve function. Recent studies (135, 136) further suggest that host circadian disruption may influence the oscillatory patterns of gut microbial composition and relative abundance, with experimental models exhibiting PMOS-like metabolic and reproductive phenotypes accompanied by alterations in gut microbiota composition and diversity. In animal studies, circadian disturbance has been associated with impaired oocyte quality and embryo developmental potential, suggesting a possible link between circadian regulation, microbiome dynamics, and reproductive dysfunction. However, current evidence is predominantly derived from observational studies and experimental animal models, and direct causal relationships between circadian disruption and follicular developmental impairment in PMOS remain insufficiently established (134, 136). Research in this area remains limited and warrants further investigation (134).

### 6.2 Gut-brain-ovary axis

The gut microbiota forms a complex bidirectional communication network with distant organs through its metabolic products and neuroimmunomodulatory capabilities. The concept of the ‘gut-brain-ovary axis’ describes a potential bidirectional communication network through which gut microbiota may influence reproductive function via immune, metabolic, endocrine, and neural pathways (125, 137). Current clinical studies have identified associations between PMOS and altered gut microbial diversity or dysbiosis; however, these findings do not yet establish whether microbiome alterations contribute directly to ovarian dysfunction or arise as a consequence of metabolic and hormonal disturbances (46, 125, 138). Emerging experimental evidence suggests that microbial metabolites, including short-chain fatty acids, bile acid derivatives, and other immunomodulatory molecules, may serve as potential mediators linking intestinal signals with ovarian function (46, 125, 138–141). Nevertheless, the precise causal pathways and key effector molecules involved in gut-brain-ovary communication remain incompletely defined and require further validation through intervention studies and integrated multi-omics analyses (125).

When gut microbiota dysbiosis occurs and Gram-negative bacteria proliferate, increased production and release of lipopolysaccharide (LPS) from their cell walls ensues (142). Concurrently, impaired intestinal barrier function due to reduced microbiota allows LPS to permeate the barrier into the portal venous circulation, triggering ‘metabolic endotoxemia’ (46, 142). Circulating LPS binds to Toll-like receptor 4 (TLR4) on ovarian tissue surfaces, activating downstream inflammatory pathways such as NF-κB. This establishes a chronic low-grade inflammatory state that subsequently suppresses granulosa cell function, promotes their apoptosis, and stimulates theca cells to produce excessive androgens (138, 143, 144). Concurrently, gut microbiota participate in bile acid metabolism and conversion (142, 143). Primary bile acids serve as natural agonists of the farnesoid X receptor (FXR) (139). Dysbiosis may alter FXR activation status, whereas FXR plays a central role in regulating glucose and lipid metabolism alongside inflammation. Dysregulation of the FXR signalling pathway may indirectly contribute to metabolic disturbances in PMOS (139–141).

The gut-brain axis constitutes a bidirectional communication system involving neural, endocrine, and immune pathways. Dysbiosis may lead to hyperactivation of the hypothalamic-pituitary-adrenal axis, increasing cortisol secretion and thereby exacerbating insulin resistance and mood disorders (145). Gut microbiota can also produce or modulate various neuroactive substances, such as gamma-aminobutyric acid and serotonin (146). These substances may influence the activity of hypothalamic kisspeptin neurons and the GnRH pacemaker via vagal or humoral pathways, thereby contributing to neuroendocrine dysregulation in PMOS (145–148).

Research on gut microbiota’s role in PMOS remains in its preliminary stages, with correlational evidence far outweighing mechanistic causal proof (125, 146, 148). Significant challenges persist in this field, including variations in dietary patterns across regions, antibiotic usage, sample collection protocols, and metagenomic analysis workflows, which have led to considerable difficulty in replicating many study findings. Future research should employ rigorously controlled prospective studies and implement standardised multi-omics integration to enhance experimental credibility and reproducibility (see **Figure 3**) (125).

**Figure 3:**
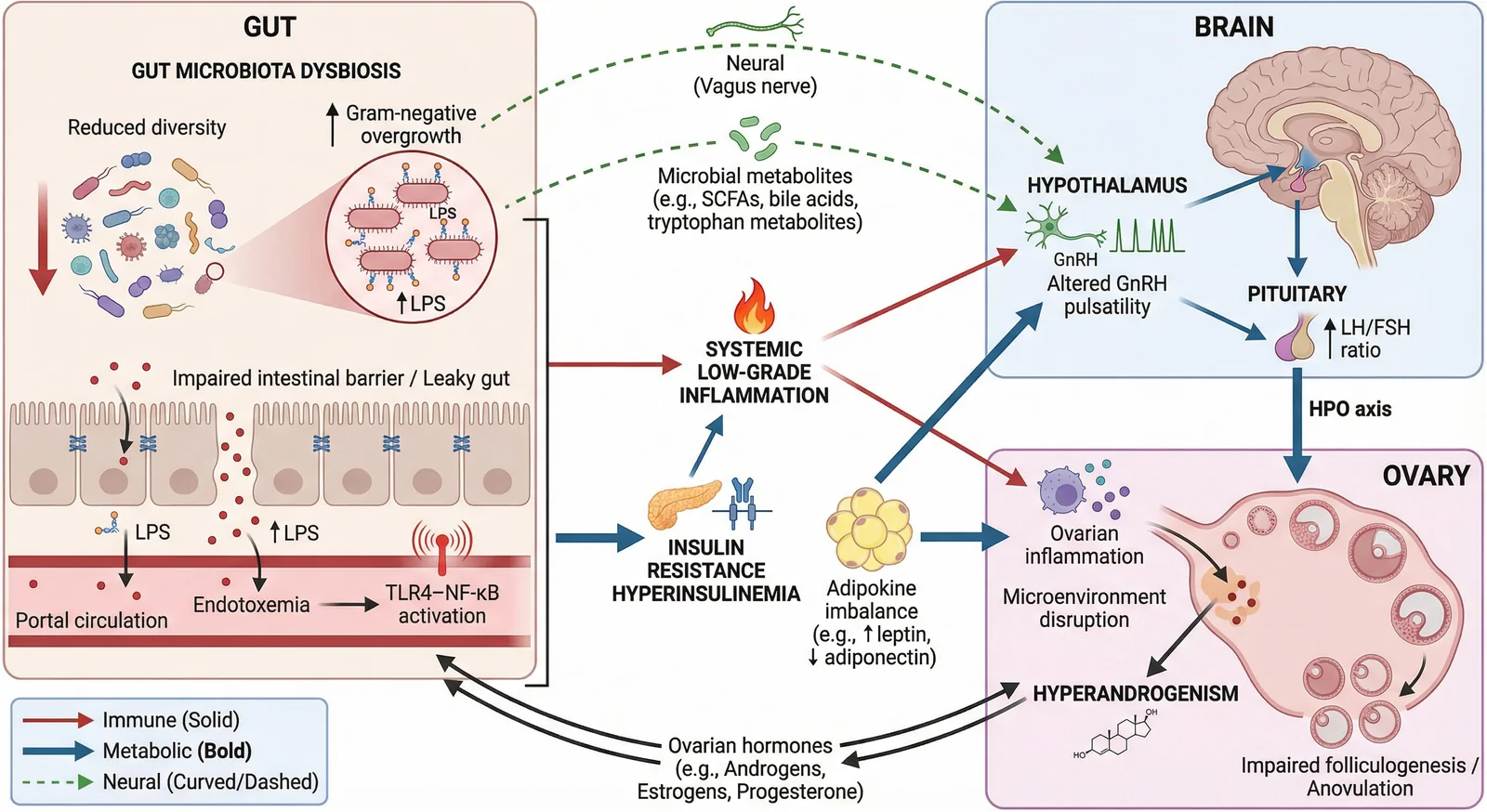
The gut-brain-ovary axis in PMOS: proposed pathways linking gut microbiota dysbiosis and metabolic endotoxemia with systemic inflammation, insulin resistance, and ovarian dysfunction. Gut microbiota dysbiosis and impaired intestinal barrier function may increase lipopolysaccharide (LPS) exposure, systemic inflammation, insulin resistance, and adipokine imbalance. Microbial metabolites and neural signals may further alter hypothalamic gonadotropin-releasing hormone (GnRH) pulsatility and hypothalamic–pituitary–ovarian signalling. These pathways converge on the ovary to promote inflammation, hyperandrogenism, impaired folliculogenesis, and anovulation. Red, blue, and dashed green arrows represent immune, metabolic, and neural/microbiota-related signalling, respectively.

## 7 Research advances and therapeutic prospects

Understanding the mechanisms underlying follicular development impairment in PMOS is shifting from the traditional ‘single-clue, single-target’ model towards a systemic cognitive paradigm characterised by ‘multi-level, networked, and dynamic’ approaches (149, 150). This transformation has been facilitated by the emergence of a series of novel research paradigms, which not only deepen our comprehension of the disease’s essence but also provide new opportunities for therapeutic development (151). Importantly, the therapeutic strategies discussed below differ substantially in their level of clinical translation. Some have been evaluated in human studies, whereas others, including several stem cell-based and inflammasome-targeted approaches, remain predominantly supported by preclinical evidence and should therefore be regarded as investigational rather than established therapeutic options (**Figure 4**).

**Figure 4:**
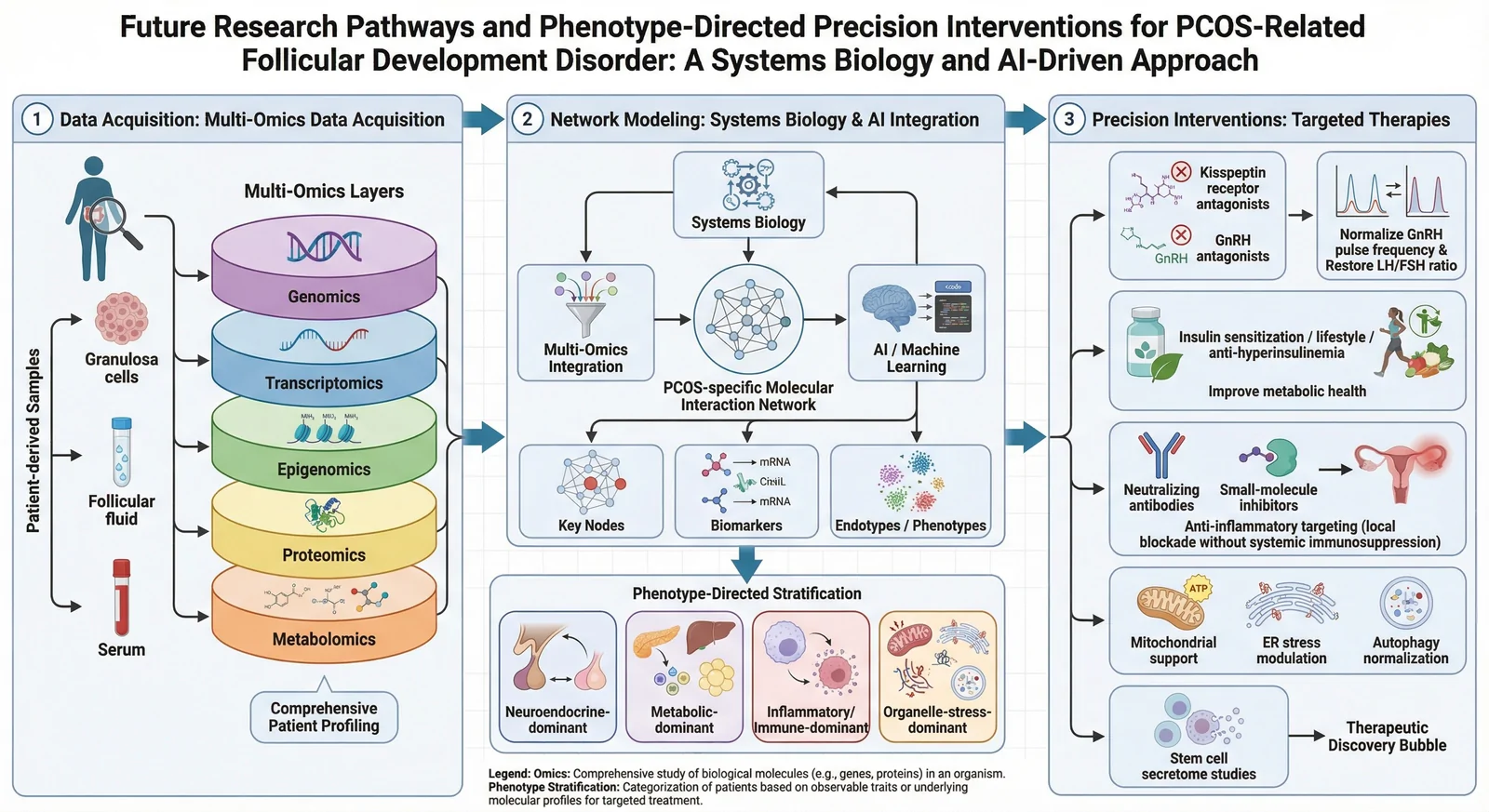
Emerging therapeutic strategies and future directions in PMOS research, integrating multi-omics, artificial intelligence, stem cell-based therapies, and targeted drug development towards phenotype-specific precision medicine. Patient-derived multi-omics data are integrated using systems biology and artificial intelligence (AI)/machine-learning approaches to identify key molecular networks, candidate biomarkers, and mechanistic endotypes. These profiles may support future stratification into neuroendocrine-, metabolic-, inflammatory/immunometabolic-, and organelle-stress-dominant patterns and facilitate evaluation of targeted therapeutic strategies. Several illustrated interventions remain investigational and are intended to represent future research directions rather than established clinical treatment algorithms.

### 7.1 Multi-omics integration and systems biology

The heterogeneous pathophysiology of polyendocrine metabolic ovarian syndrome (PMOS) is unlikely to be fully explained by a single molecular pathway. Recent multi-omics studies integrating transcriptomics, proteomics, metabolomics, lipidomics, and phosphoproteomics have identified alterations in metabolic regulation, mitochondrial function, steroidogenesis, inflammatory signalling, and follicular-cell activity in women with PCOS/PMOS (149, 152–154). These findings provide additional insight into the molecular abnormalities associated with impaired follicular development.

For example, integrated follicular-fluid metabolomics and cumulus-cell transcriptomics revealed alterations in the tricarboxylic acid cycle, oxidative phosphorylation, cAMP signalling, mTOR signalling, and steroid-hormone biosynthesis in women with PCOS (149). Proteomic and lipidomic studies have also identified abnormalities in lipid metabolism, immune regulation, and granulosa-cell signalling (152–154). However, most available studies are based on relatively small and cross-sectional cohorts. Therefore, the identified molecular signatures should currently be considered exploratory associations rather than established causal mechanisms or clinically validated biomarkers.

### 7.2 Artificial intelligence and machine learning

Artificial intelligence (AI) and machine-learning approaches are increasingly being investigated for the diagnosis, phenotypic classification, and reproductive-outcome prediction of PMOS. Recent studies have explored AI-assisted ovarian ultrasound assessment, prediction of IVF outcomes, and identification of patients at increased risk of impaired oocyte maturation (155–157). These studies suggest that AI may complement conventional clinical and laboratory parameters and potentially support individualized reproductive management.

Nevertheless, the clinical application of AI in PMOS remains at an early stage. Current models are frequently limited by relatively small datasets, retrospective designs, class imbalance, and insufficient external validation (155). Accordingly, AI should presently be regarded as a decision-support and research tool rather than an established method for autonomous diagnosis or treatment selection. Future multicentre and prospective studies are required to determine whether these models improve clinically meaningful outcomes.

### 7.3 Stem cell therapy

Given the chronic inflammation, fibrosis, and oxidative stress present in PMOS ovaries, stem cell-based approaches have emerged as a potential experimental strategy for modulating the ovarian microenvironment (158). Current research primarily focuses on mesenchymal stem cells (MSCs), whose proposed therapeutic effects do not depend on oocyte differentiation. Instead, MSCs appear to exert paracrine effects through the secretion of multiple bioactive factors, thereby contributing to anti-inflammatory and immunomodulatory responses, inhibition of fibrosis, promotion of angiogenesis, and anti-apoptotic effects (159). In animal models (160), transplanted MSCs have been reported to improve reproductive cyclicity, reduce the number of follicular cysts, and promote dominant follicle development and ovulation.

However, the evidence supporting MSC-based interventions in PMOS remains predominantly preclinical, and these findings should not be interpreted as evidence of established clinical efficacy. Although early translational investigations may support further clinical evaluation, the optimal cell source, administration route, dosage, treatment schedule, long-term efficacy, and reproductive safety remain uncertain. Therefore, MSC-based therapy should currently be regarded as an investigational approach rather than an established treatment for PMOS, and its clinical value requires confirmation in well-designed clinical trials. Furthermore, cell-free therapeutic strategies based on MSC-derived secretomes or extracellular vesicles may represent another potential research direction, but these approaches likewise remain experimental and require further preclinical and clinical validation.

### 7.4 Novel approaches to targeted drug development

With an increasingly detailed understanding of the molecular networks underlying PMOS, therapeutic strategies are gradually shifting from empirical ovulation induction toward mechanism-based precision interventions. Among the potential targets, dysregulation of the neuroendocrine axis represents a particularly important upstream therapeutic opportunity. Women with PMOS frequently exhibit increased GnRH and LH pulse frequencies, resulting in relative LH hypersecretion, insufficient FSH support, and enhanced androgen production by ovarian theca cells. Persistent exposure to excessive androgens, together with inadequate FSH stimulation, may impair granulosa-cell aromatase activity and responsiveness to FSH, disrupt dominant follicle selection, and ultimately perpetuate follicular developmental arrest (18). Therefore, targeting the kisspeptin–KISS1R–GnRH axis has a clear pathophysiological rationale.

Kisspeptin is a major upstream regulator of GnRH neurons. In a mouse model of PMOS, selective inhibition of endogenous kisspeptin neurons reduced the abnormally elevated LH pulse frequency and reversed hyperandrogenaemia, providing preclinical evidence that suppression of excessive kisspeptin-mediated neuroendocrine drive may have therapeutic potential (161). Furthermore, a randomized, placebo-controlled phase II trial of the neurokinin 3 receptor antagonist AZD4901, involving 67 women with PMOS, demonstrated reductions in LH pulse frequency, cumulative LH exposure, and serum testosterone concentrations (162). However, this short-term study primarily evaluated neuroendocrine and hormonal endpoints and was not adequately powered to determine improvements in sustained ovulation, pregnancy, or live-birth outcomes. These findings therefore support the feasibility of pharmacologically modulating the KNDy neuronal network in humans, but its clinical reproductive efficacy remains to be established.

Nevertheless, kisspeptin-targeted therapy should not be equated with universal KISS1R antagonism. Previous studies have shown that repeated administration of kisspeptin-54 may promote dominant follicle development or induce ovulation in a subset of women with anovulatory PMOS, although the overall response has been inconsistent (163). Importantly, the available human evidence is based on a very small proof-of-concept study involving only 12 women, and follicular development and ovulation were observed only in a subset of participants. Thus, these findings cannot yet establish reliable effects on overall ovulation rates, pregnancy, or live birth. These observations suggest that the kisspeptin pathway may have phenotype-dependent and potentially bidirectional therapeutic effects. In patients characterized by excessive kisspeptin/KNDy neuronal activity and increased LH pulsatility, reducing the activity of this pathway may be more appropriate. Conversely, in selected patients with preserved pituitary–ovarian responsiveness but insufficient endogenous gonadotropin stimulation, exogenous kisspeptin administration may also offer therapeutic value. Accordingly, KISS1R antagonists should currently be regarded as mechanistically supported preclinical or early translational candidates. Their optimal target population, dosing regimen, pharmacological selectivity, and effects on physiological gonadotropin secretion and reproductive outcomes remain to be established.

Recent evidence further suggests that kisspeptin may contribute to both central neuroendocrine abnormalities and local ovarian dysfunction in PMOS. A prospective cohort study published in 2026 found that women with PMOS had higher serum and follicular-fluid kisspeptin concentrations during controlled ovarian stimulation than women without PMOS. Kisspeptin concentrations were positively associated with the number of retrieved oocytes but negatively associated with the proportion of high-quality embryos (164). In addition, a translational study showed that excessive kisspeptin exposure aggravated androgen-induced oxidative stress, energy-metabolism impairment, and apoptosis in granulosa cells by disrupting Drp1 phosphorylation and promoting excessive mitochondrial fission (165). These studies primarily provide associative or mechanistic evidence and do not establish the clinical efficacy of KISS1R-targeted pharmacotherapy. Nevertheless, they highlight the need for future studies to distinguish the central neuroendocrine effects of kisspeptin from its local ovarian actions and to select the direction and therapeutic window of intervention according to individual neuroendocrine and ovarian phenotypes.

Clinical evidence regarding GnRH receptor antagonists remains inconsistent. In a randomized, double-blind phase II trial involving 114 women with PMOS, elagolix reduced circulating LH, oestradiol, and testosterone concentrations but did not significantly restore regular menstrual cycles (166). Only three of the 114 participants met the prespecified primary endpoint of menstrual-cycle normalization, including two participants receiving placebo and one receiving elagolix. Thus, despite clear endocrine effects, this study did not demonstrate a corresponding improvement in clinically meaningful reproductive function, and pregnancy and live-birth benefits were not established.

By contrast, a 2026 study integrating experiments in a PMOS-like mouse model with a phase I clinical trial evaluated low-dose GnRH receptor antagonism with ganirelix (167). In the human study, 18 evaluable women with PMOS without obesity received a single low dose of ganirelix during the early follicular phase at either 0.025 or 0.0625 mg. Both doses reduced LH pulse frequency, mean LH concentrations, and the LH-to-FSH ratio, while serum androstenedione concentrations decreased by approximately 25–30% without marked suppression of oestradiol. In the animal model, intermittent low-dose treatment also improved oestrous cyclicity and ovulation and reduced androgen levels (167). However, the human component was limited by the small sample size, single-dose design, and short-term assessment of neuroendocrine and hormonal endpoints. Sustained ovulation, pregnancy, and live-birth outcomes were not evaluated. Therefore, although low-dose GnRH receptor antagonism represents a promising strategy for partially recalibrating excessive GnRH/LH signalling without complete gonadotropin suppression, its reproductive efficacy requires confirmation in larger, longer-term randomized trials.

In summary, targeting the kisspeptin–KISS1R–GnRH/KNDy network offers a biologically plausible approach to correcting upstream neuroendocrine abnormalities in PMOS.However, the available human evidence remains limited to relatively small proof-of-concept or early-phase studies, most of which have focused on hormonal and neuroendocrine surrogate endpoints. At present, there is no robust evidence that kisspeptin/KISS1R-, neurokinin-B/NK3R-, or GnRH receptor-targeted therapies improve sustained ovulation, clinical pregnancy, or live-birth rates. Larger, phenotype-stratified randomized trials with clinically meaningful reproductive endpoints are therefore required before these approaches can be incorporated into routine fertility treatment.

In addition to central neuroendocrine modulation, improvement of the local inflammatory and immune microenvironment within the ovary represents another important direction for targeted drug development (168–170). Persistent low-grade inflammation, immune-cell imbalance, and aberrant cytokine production in the PMOS ovary may disrupt granulosa cell–oocyte communication, mitochondrial function, and follicular homeostasis. Accordingly, proinflammatory mediators such as TNF-α, IL-1β, and IL-6, as well as inflammatory and pyroptotic signalling pathways including TLR4/MyD88/NF-κB and NLRP3/caspase-1/GSDMD, may represent potential targets for modifying the ovarian microenvironment. In recent years, inhibition of NLRP3-mediated granulosa-cell pyroptosis has shown potential to ameliorate ovarian inflammation, endocrine disturbances, and impaired follicular development in animal models of PMOS (168–170). However, the current evidence is derived predominantly from cellular and animal studies and remains insufficient to establish that neutralizing antibodies or small-molecule inhibitors directed against individual inflammatory mediators can improve ovulation or fertility outcomes in women with PMOS. Future strategies integrating ovary-targeted drug delivery, patient-phenotype stratification, and reliable pharmacodynamic biomarkers may enhance local therapeutic efficacy while reducing systemic exposure. Nevertheless, the pharmacokinetic characteristics, off-target effects, reproductive safety, and long-term risks of these approaches require systematic evaluation.

### 7.5 Clinical implications and precision stratification

The multidimensional model proposed in this review may provide a mechanistic framework for refining patient stratification beyond conventional clinical phenotypes. Rather than representing mutually exclusive subtypes, patients with PCOS may exhibit overlapping neuroendocrine-, metabolic-, inflammatory/immunometabolic-, or organelle-stress-dominant features. Integrating these mechanistic characteristics with conventional reproductive and metabolic phenotypes may help identify biologically distinct endotypes and improve understanding of the marked clinical heterogeneity of PCOS.

Such stratification may also facilitate prediction of treatment response. For example, patients with prominent insulin resistance and hyperinsulinemia may be more likely to benefit from metabolic optimization, whereas neuroendocrine-dominant or inflammatory phenotypes may provide a rationale for future pathway-specific interventions. However, these mechanistic profiles are currently conceptual and should not yet be regarded as validated treatment-selection criteria.

From a biomarker perspective, the network model suggests that composite signatures may be more informative than individual markers. Routinely available endocrine and metabolic parameters could be combined with emerging inflammatory, granulosa-cell, follicular-fluid, microbiome-related, and organelle-stress markers identified through multi-omics approaches. Prospective studies are required to determine whether such multimarker panels can predict ovulatory recovery, response to treatment, or oocyte developmental competence and provide additional value beyond conventional clinical variables.

## 8 Summary and outlook

This review systematically delineates a multilayered and networked pathophysiological landscape of follicular development impairment in PMOS. Current evidence suggests that early-life genetic susceptibility and epigenetic programming—potentially shaped by environmental factors such as intrauterine hyperandrogenism—may interact with post-pubertal dysregulation of two major systems, neuroendocrine and metabolic regulation. These systemic abnormalities may promote a chronic inflammatory and immune-dysregulated state within the local ovarian microenvironment, accompanied by impaired granulosa–oocyte communication and disturbances in cellular and organelle homeostasis. However, important limitations of the current evidence base should be acknowledged. Although several of these abnormalities have been demonstrated in human PMOS tissues or patient-derived cells, mechanistic and causal evidence linking specific pathways—including gap-junction dysfunction, mitochondrial impairment, immune-cell remodelling, and fibrosis—to follicular arrest remains derived predominantly from animal and cellular models. Their effects may be further modulated by emerging pathways such as the gut–brain–ovary axis, although the causal contribution of these pathways remains to be fully established. Collectively, these interacting abnormalities may contribute to widespread follicular arrest and impaired ovulation.
